# Testicular self‐examination: The role of anticipated relief and anticipated regret

**DOI:** 10.1111/bjhp.12756

**Published:** 2024-09-29

**Authors:** Sara Lorimer, Teresa McCormack, Christoph Hoerl, Sarah R. Beck, Matthew Johnston, Aidan Feeney

**Affiliations:** ^1^ School of Psychology Ulster University Coleraine UK; ^2^ School of Psychology Queen's University Belfast Belfast UK; ^3^ Department of Philosophy University of Warwick Coventry UK; ^4^ School of Psychology University of Birmingham Birmingham UK; ^5^ School of Philosophy, Psychology and Language Sciences University of Edinburgh Edinburgh UK

**Keywords:** counterfactual thinking, decision‐making, emotions, health promotion, intention, regret, relief, testicular self‐examination

## Abstract

**Objective:**

Anticipated regret has been implicated in health‐related decision‐making. Recent work on influenza vaccination has suggested that anticipated relief, too, may influence individuals' decisions to engage in positive health behaviours. To explore these affective components further and address the generality of possible mechanisms underlying these associations, we examined whether anticipated relief and anticipated regret independently predict testicular self‐examination (TSE) intention and behaviour. Given claims about differences in their nature and function, we distinguished between counterfactual relief (relief that a worse outcome did not obtain) and temporal relief (relief that an unpleasant experience is over).

**Design:**

Prospective correlational.

**Methods:**

At Time 1 (July 2022), 567 cis‐gendered males were asked to complete measures of anticipated regret, anticipated counterfactual and temporal relief, measures of the Theory of Planned Behaviour and measures of anxiety and shame. One month later, the same participants were recontacted and asked about their engagement in TSE in the previous month.

**Results:**

Anticipated counterfactual relief and anticipated regret are independent, positive, predictors of intention to engage in TSE and, indirectly, TSE behaviour itself. Interestingly, anticipated temporal relief was negatively associated with intention to engage in TSE and, indirectly, behaviour.

**Conclusions:**

Our results suggest that it may be the counterfactual nature of anticipated regret and anticipated relief that underlies their positive association with TSE and other health‐promoting behaviours. Interventions designed to increase engagement in preventive health behaviours, such as TSE, may benefit from the consideration of both positively and negatively valenced counterfactual emotions.

Decisions to engage in healthful behaviours are known to be associated with emotions anticipated to arise from those decisions. Particular attention has been paid to the anticipation of regret; a counterfactual emotion triggered by the realization that a different decision would have led to a better outcome (see Landman, 1993). Typically, the extent to which individuals anticipate that they will regret not engaging in a particular behaviour is positively associated with their intention to engage in that same behaviour. For example, in the case of exercise, greater anticipated regret for not exercising is directly, and positively, associated with intention to do so, and indirectly, through intention, associated with actual exercise behaviour in subsequent weeks (Abraham & Sheeran, 2003, 2004). Beyond exercise, anticipated regret is known to predict a variety of healthful behaviours including vaccine uptake and cancer screening (Chapman & Coups, 2006; Conner et al., 2006; Hamilton & Schmidt, 2014; Lazuras et al., 2012; see Brewer et al., 2016 for meta‐analytical review of 81 studies). However, the reason for this association is unclear. Specifically, it may be the negative valence of regret that underlies the association. This explanation is commonly offered by behavioural scientists who suggest that, given the aversive nature of regret, there is a drive to behave in ways that will minimize or eliminate the possibility of experiencing future regret (e.g., Zeelenberg & Pieters, 2007). Alternatively, it may be regret's counterfactual nature that is the important factor; the process of considering future regret may require individuals to simulate and compare a number of action–outcome combinations, along with their associated affective states.

A deeper understanding of the association between anticipated regret and engagement in healthful behaviour is likely to be important if we wish to make use of anticipated regret, and other pertinent emotions, in the design of effective health interventions. Indeed, there has recently been a call for health interventions and behaviour change strategies to be more precisely defined in terms of specifying how they are believed to work, for whom and under what conditions (Rothman & Sheeran, 2021). Furthering our understanding of the link between anticipated regret and behaviour seems particularly important given that interventions that have utilized anticipated regret have produced mixed results (e.g., Cox et al., 2014; O'Carroll et al., 2011, 2016). So far, little exists in the way of explaining the observed variability in intervention outcomes.

Recent research on relief suggests a way forward in understanding the respective roles of valence and counterfactual thinking in health‐related decision‐making. In the literature on relief, authors have suggested a distinction between relief experienced upon realizing that a different decision would have resulted in a worse outcome and relief that an unpleasant experience is over (Graham et al., 2023; Hoerl, 2015; Lorimer et al., 2022; Sweeny & Vohs, 2012). Following the nomenclature of Hoerl (2015), we will refer to these as *counterfactual relief* and *temporal relief*, respectively. Anticipated counterfactual relief is interesting in the current context because it is a positive counterfactual emotion, arising from downward counterfactual thinking (‘if I had done X, things would have been worse’), as opposed to upward counterfactual thinking that leads to regret (‘If I had done Y, things would have been better’). If the predictive power of regret lies in its negative valence, a measure of the relief that people anticipate experiencing following healthful behaviour should not predict that behaviour. On the other hand, if it is regret's counterfactual basis which determines its predictiveness, then a measure of anticipated counterfactual (but not temporal) relief might also predict behaviour in addition to or instead of a measure of anticipated regret.

Applying this analytic framework to decisions about influenza vaccination, Lorimer et al. (2023) asked participants to anticipate the regret they might feel if they did not get vaccinated against influenza and the relief they would feel if they did. Participants anticipated the relief they would experience both if they decided to get the vaccination rather than deciding not to (counterfactual relief) and, separately, at the end of the vaccination appointment (temporal relief). Whereas anticipated regret and anticipated counterfactual relief independently predicted intentions to get vaccinated and—indirectly—subsequent vaccination status, anticipated temporal relief did not predict vaccination intention. This result suggests that it is the counterfactual nature rather than valence of regret which underlies its predictive power, whilst the independence of anticipated regret and anticipated counterfactual relief as predictors may be attributable to the difference in direction (upward or downward) of the counterfactual thought which they result from.

These findings help us to specify the reasons why anticipating regret predicts health‐related decision‐making, but further work is necessary to specify the operating conditions (Rothman & Sheeran, 2021) for anticipated emotion interventions. In particular, anticipated regret (and relief) might work differently for different decision contexts. Although anticipated regret has been most often studied in the context of decisions about vaccination (see Bonner et al., 2023; Fung et al., 2023; Ziarnowski et al., 2009), there have also been studies of its role in other health‐promoting decisions such as cancer screening (e.g., Hunkin et al., 2020; O'Carroll et al., 2015; Sandberg & Conner, 2009) and safe sex/condom use (e.g., Abraham et al., 2004; Bakker et al., 1997). These decisions differ in several respects (see Brewer et al., 2016) including the severity of the consequences that might arise should the decision be not to act, the interval between the decision and its consequences becoming known, and the frequency of the behaviour. Another feature which has not been considered is the nature of the health‐promoting behaviour itself and when it might be said to have ended. This is particularly important to conclusions about the role that might be played by anticipated temporal relief (relief at the end of an aversive experience) in health‐promoting decisions. For example, participants in Lorimer et al.'s (2023) study were asked to anticipate the relief they would experience at the end of their vaccination appointment. However, because a vaccination can result in unpleasant symptoms for several days after it has been administered (e.g., see Nichol et al., 1996), participants may not have been anticipating temporal relief at the end of the vaccination appointment. This might explain why temporal relief did not predict intentions or decisions to avail of vaccination in Lorimer et al.'s study. Thus, in the absence of a study generalizing its findings to a different health‐promoting decision, Lorimer et al.'s (2023) findings may not be unequivocally interpreted as evidence that it is the counterfactual nature of regret and relief which underlies their association with health‐promoting decisions.

To investigate the generality of Lorimer et al.'s findings, in the study to be described below, we focused on decisions about testicular self‐examination (TSE) in men. Testicular cancer is the most common cancer in young men (Znaor et al., 2014), with incidence rates increasing globally (Shanmugalingam et al., 2013) and highest in Europe, North America and Australia (Park et al., 2018). Although mortality rates associated with testicular cancer are low, early detection of the disease is crucial for timely treatment and improved patient outcomes (Huyghe et al., 2007). Recommendations regarding TSE are mixed with some organizations suggesting that the cost, both financial and emotional, of false positives outweighs the benefits of early detection of a largely treatable disease (see Chong et al., 2023 for summary). Indeed, the US Preventive Services Task Force recommends against TSE (USPSTF, 2011). On the other hand, regular engagement in TSE has been suggested as an easy and effective form of detection that can facilitate early diagnosis (Ugurlu et al., 2011). Thus, encouraging monthly TSE has been a focus of numerous UK National Health Service Trusts and cancer prevention charities worldwide (e.g., American Cancer Society, Testicular Cancer Society).

Despite the potential importance of TSE and the limited practical barriers restricting engagement, rates of TSE are low (Evans et al., 2006; Peltzer & Pengpid, 2015) and research exploring the factors that influence decisions to perform TSE is sparse. In what does exist, the Theory of Planned Behaviour (TPB; Ajzen, 1998) is commonly used to explain variance in TSE intention and behaviour (Iyigun et al., 2016; McGilligan et al., 2009). The TPB is a theoretical framework used to explain and predict behaviour in a variety of contexts and is often used as a basis on which to build behaviour change interventions (see Steinmetz et al., 2016 for meta‐analytical review). Briefly, the TPB suggests that people's attitudes towards a certain behaviour (attitudes), their beliefs that the behaviour is socially approved or expected (subjective norms) and beliefs that they are capable of the behaviour (perceived control) influence the formation of behavioural intention. Intention, in turn, is the direct and most proximal predictor of behaviour itself (see Hagger & Hamilton, 2024 for overview and meta‐analytical review). The TPB has been shown to account for more variance in TSE behaviour than other popular models of behaviour including the Health Belief Model (McClenahan et al., 2007). Whilst the TPB variables, in and of themselves, are often explored as predictors of specific behaviours, it is also common practice for the TPB to be ‘extended’ by including anticipated regret as an additional, affective, predictor of intention. In the context of TSE, McClenahan et al. (2007) found that anticipated regret accounted for significant additional variance in TSE intentions once other TPB variables had been accounted for.

Decisions about whether to carry out TSE are very different to the vaccination decisions studied by Lorimer et al. (2023). First, where TSE is recommended, it is recommended to be carried out frequently (i.e., every month) whereas decisions about influenza vaccination need be made only once a year. Second, the potential consequences of deciding not to self‐examine include death from testicular cancer or loss of a testicle, whereas the prototypical outcomes of influenza infection are likely much less severe. Third, consequences of a decision about influenza vaccination will be known within 6 months at most (the influenza season in which the decision is taken) whereas consequences of decisions about TSE may not be known for years. Finally, and most important with respect to the potential role of anticipated temporal relief, a negative testicular self‐examination ends when the examination ends, and thus temporal relief may be anticipated at that point. In the case of influenza vaccination, participants may anticipate negative symptoms in the days following the vaccination and thus may not anticipate temporal relief at the end of the vaccination appointment.

Shepherd et al. (2017) explored the social, cognitive and emotional predictors of TSE, including measures of anticipated emotion. Anticipated relief—the extent to which one would feel relieved having performed TSE—but not anticipated regret—the extent to which one would feel regret not having performed TSE—predicted TSE intention and past TSE behaviour. This finding held even when TPB constructs, and anxiety and shame associated with a potential cancer diagnosis, were statistically controlled for. One limitation of the study is that the researchers did not distinguish between temporal and counterfactual relief, thus it is not clear whether the relief reported by participants relates to a positive emotion anticipated (a) because of a decision to engage in the self‐examination rather than deciding not to (i.e., counterfactual relief), or (b) upon completion of a potentially aversive (Miller et al., 2022) self‐examination (i.e., temporal relief). Accordingly, the results of the study cannot be used to evaluate claims about the role played by the counterfactual nature of regret and relief in their association with healthful intentions and behaviour. A second limitation of this study is that only intention was assessed, without exploring subsequent behaviour, leaving it undetermined if—and which—variables predicted behaviour directly. We seek to address this within the current study by assessing both behavioural intention and actual behaviour.

## THE CURRENT STUDY

In the present study, we examine whether and how anticipated temporal relief, counterfactual relief and regret relate to TSE intention and behaviour when controlling for other well‐established predictors of healthful behaviour. Given that Shepherd et al. found that anticipated regret did not predict TSE intention, the anticipation of counterfactual emotions may play no role in decisions concerning TSE. If this were to be the case, we would predict that only temporal relief is associated with TSE intention. This possibility is supported by theorizing on the function of temporal relief, which suggests that the anticipation of temporal relief may encourage engagement in aversive, but ultimately beneficial behaviours (Hoerl, 2015). An alternative possibility is that it is the counterfactual nature of regret and relief that underlies their association with healthful behaviours. In this case, we would predict either anticipated counterfactual relief, regret, or both‐ but not anticipated temporal relief‐ to be associated with TSE intention. A final possibility is that specifically negative anticipated emotions predict healthful behaviours. Although this is contrary to Shepherd et al.'s results, it does align with the extant regret literature that links decision‐making to a desire to minimize regret (Loomes & Sugden, 1982; Zeelenberg & Pieters, 2007). If this were the case, we would predict that anticipated regret but neither measure of relief predicts TSE intentions.

## METHOD

### Design

The study adopted a prospective correlation design with variables measured across two time points. Anticipated emotions (regret, counterfactual relief, temporal relief), TPB constructs (attitudes, subjective norms and perceived behavioural control), control variables (shame and anxiety) and TSE intention were measured at Time 1. Actual TSE behaviour was assessed at Time 2, one month after the initial survey.

### Participants

At Time 1, 567 cisgendered males (*M*_age_ = 39.6 years, SD_age_ = 12.9 years) from the United Kingdom were recruited online using Prolific (https://www.prolific.co). Sample size was based on the requirements for a binary logistic regression that was used to analyse the Time 2 data. This analysis required 180 participants in the smallest cell of the outcome variable to comply with a recommendation of 20 observations per predictor variable (Austin & Steyerberg, 2017). Based on Shepherd et al. (2017), we expected ~43% of participants to have performed a testicular self‐examination between Time 1 and Time 2 meaning that we required 419 participants in total for the Time 2 analysis. To account for an attrition rate of ~30% (based on our previous studies using a one‐month follow‐up period on Prolific), and potentially missing or incomplete data, this number was rounded to a target sample size of 560 participants. In total, 567 participants were recruited. Participants received £1.00 for taking part at Time 1, and £.30 at Time 2. 82.0% of participants (*n* = 465, *M*_age_ = 40.7 years, SD_age_ = 12.9 years) returned at Time 2. There were no differences in any Time 1 measures between those who did and did not return at Time 2. Ethical approval was granted by the Research Ethics Committee of the last author's university.

### Materials and procedure

Questionnaires (see Appendix S1 for exact phrasing of all questions) were administered online using Qualtrics (https://www.qualtrics.com/uk/). At Time 1, participants provided demographic information, and their beliefs about how often TSE should be performed were assessed. Participants then answered four questions related to each of the TPB variables: attitudes (e.g., ‘Performing testicular self‐examination within the next month would be…’ with a 7‐point response option ranging from a negative attitude, e.g., ‘harmful’, to a corresponding positive attitude, e.g., *‘*beneficial’; α = .67, increased to *α* = .77 with the removal of ‘pleasant’ item), subjective norms (e.g., ‘Most people who are important to me think I should perform testicular self‐examination within the next month’; *α* = .83) and perceived behavioural control (e.g., ‘I am confident that I could perform a testicular self‐examination within the next month’; *α* = .71). Responses to the subjective norms and perceived behavioural control questions were given on a 7‐point scale from ‘strongly disagree’ to ‘strongly agree’. Questions were formulated using Francis et al.'s (2004) TPB questionnaire manual.

Following this, participants were asked two questions concerning anticipated temporal relief (e.g., *If I performed a testicular self‐examination within the next month*, *I would feel relieved once it was over*; *α* = .94), anticipated counterfactual relief (e.g., *If I performed a testicular self‐examination within the next month*, *rather than deciding not to*, *I would feel relieved*; *α* = .87) and anticipated regret (e.g., *If I did not perform a testicular self‐examination within the next month*, *rather than deciding to do it*, *I would feel regret*; *α* = .87). The relationship between these questions, adapted from Lorimer et al. (2023), and those used by Shepherd et al. is considered in supplementary materials. All responses to questions described thus far were given on a 7‐point scale from ‘strongly disagree’ to ‘strongly agree’. In line with Shepherd et al. (2017), nine questions were used to assess anticipated shame associated with a testicular cancer diagnosis (e.g., *To what extent would you feel ashamed if you were diagnosed with testicular cancer? α* = .94) and experienced anxiety associated with testicular cancer and associated medical procedures (e.g., *To what extent are you anxious that you may have testicular cancer? α* = .86). Responses to these questions were given on a 7‐point scale from ‘not at all’ to ‘extremely’.

Finally, participants were asked three questions assessing their intention to perform a testicular self‐examination within the next month (e.g., *I intend to perform a testicular self‐examination in the next month; α* = .96), and additionally whether they had performed a testicular self‐examination in the past month. Responses to the intention questions were given on a 7‐point scale from ‘strongly disagree’ to ‘strongly agree’. At Time 2, one month later, the same participants were recontacted and asked whether they had performed a testicular self‐examination in the last month; ‘Have you performed a testicular self‐examination in the last month?’ with a binary ‘*Yes’* or ‘*No’* response option. To facilitate analysis, ‘No’ and ‘Yes’ responses were coded as 0 and 1, respectively.

## RESULTS

At Time 1, 27.9% of participants had performed a testicular self‐examination within the past month. Fifty‐three percent of participants indicated that they believed TSE should be performed every month, whilst 35.5% believed that it should be performed less frequently. Means, standard deviations and intercorrelations of all key variables are provided in Table 1; the results indicate that anticipated temporal relief, anticipated counterfactual relief and anticipated regret are related to intention to perform testicular self‐examination and actual behaviour at Time 2.

**TABLE 1:** Means, standard deviations and intercorrelations of key variables.

	*M* (SD)	1	2	3	4	5	6	7	8	9
1. ATR	4.97 (1.39)	–								
2. ACF	4.87 (1.40)	.42***	–							
3. AReg	3.42 (1.56)	.31***	.52***	–						
4. Attitudes	6.0 (1.07)	.095*	.38***	.16***	–					
5. Subjective norms	3.44 (1.38)	.20***	.45***	.51***	.29***	–				
6. Perceived control	6.08 (.95)	−.002	.32***	−.002	.47***	.21***	–			
7. Anxiety	3.10 (1.13)	.17***	.10*	.28***	−.090*	.14***	−.22***	–		
8. Shame	3.03 (1.57)	.066	−.10*	.032	−.22***	−.084*	−.29***	.47***	–	
9. Intention	4.89 (1.75)	.13**	.59***	.48***	.43***	.55***	.48***	.11**	−.17***	–
10. Examination	NA	.046	.27***	.28***	.12*	.30***	.27***	.020	−.088	.49***

*Note*: Examination refers to testicular self‐examination performed between Time 1 and Time 2. All variables were measured on a scale from 1 to 7, except examination which is binary coded with non‐examination as reference (0). *N* = 567 for all associations except associations with examination where *N* = 465.

Abbreviations: ACF, anticipated counterfactual relief; AReg, anticipated regret; ATR, anticipated temporal relief.

***p < .001, **p < .01, *p < .05.

### Predicting TSE intention

A two‐step linear regression was used to determine the predictors of TSE intention. The full model can be seen in Table 2. Where the primary focus is extension of the TPB model, it is common to enter anticipated emotion variables after norms, attitudes and perceived control. Because our focus is on anticipated emotion, we followed the approach of Lorimer et al. (2023) by first entering the anticipated emotion variables and only then entering the TPB variables. This approach allows us to examine the predictive power of the anticipated emotion variables on their own and to test hypotheses about which anticipated emotions would best predict behavioural intentions. Thus, anticipated temporal relief, anticipated counterfactual relief and anticipated regret were entered at Step 1, and the TPB variables, anxiety and shame, were entered at Step 2. When entered at Step 1, the three anticipated emotion variables accounted for 42% of the variance in intention, representing a significant improvement compared to a chance model. At this step, both anticipated counterfactual relief and anticipated regret were positive predictors of intention whereas anticipated temporal relief was a negative predictor. The remaining predictors of intention were added at Step 2 resulting in further improvement to the model. With all predictors included, 58% of the variance in intention was accounted for. All predictors in the final step were significant; anticipated counterfactual relief, anticipated regret, TPB variables and anxiety were all positive predictors of intention, whereas anticipated temporal relief and anticipated shame were negative predictors of intention.

**TABLE 2:** Summary of hierarchical regression models predicting testicular self‐examination intention and behaviour.

	Intention				Behaviour			
	Step 1		Step 2		Step 1		Step 2	
	*B* (SE)	*β*	*B* (SE)	*β*	*B* (SE)	OR	*B* (SE)	OR
Temporal relief	−.23 (.05)	−.18***	−.16 (.04)	−.13***	−.17 (.08)	.85*	−.02 (.09)	.99
Counterfactual relief	.67 (.05)	.53***	.36 (.05)	.29***	.35 (.09)	1.42***	−.01 (.12)	.99
Regret	.29 (.04)	.26***	.24 (.04)	.21***	.28 (.08)	1.32***	.08 (.10)	1.08
Attitudes			.16 (.05)	.095**			−.39 (.14)	.68**
Norms			.29 (.04)	.23***			.17 (.10)	1.19
Control			.55 (.06)	.30***			.38 (.16)	1.46*
Anxiety			.19 (.05)	.12***			−.07 (.12)	.93
Shame			−.074 (.04)	−.067*			.04 (.08)	1.05
Intention							.63 (.10)	1.87***
*R* ^2^	.42		.58		.14		.34	
*F* change	134.9***		45.9***					
Step *χ* ^2^					52.5***		84.1***	

*Note*: Nagelkerke *R* ^2^ is reported for behaviour regression.

Abbreviation: OR, odds ratio.

***p < .001, **p < .01, *p < .05.

### Predicting TSE behaviour

By Time 2, 53.3% of participants had performed TSE. To establish the predictors of behaviour, a two‐step binary logistic regression was used. As shown in Table 2, the two‐step model followed a similar structure to that used in the prediction of intention, except intention itself was added alongside the other predictors at Step 2. At Step 1, 14% of the variance in behaviour was accounted for. The model at this step was a significantly better fit for the data compared to a model based on chance. At this step, both anticipated counterfactual relief and anticipated regret were positive predictors of behaviour, whereas anticipated temporal relief was a negative predictor of behaviour. The remaining predictors, including intention, were added to the model at Step 2. This resulted in further improvement to the model with 34% of the variance in TSE behaviour accounted for. At this step, only attitudes, perceived control and intention were significant predictors of behaviour; more favourable attitudes were associated with a reduced likelihood of engaging in TSE, whereas a greater sense of control and higher intentions were associated with higher likelihood of performing TSE.

When considered together, the correlations and linear regression indicate a relation between each of three anticipated emotions, and intention and TSE behaviour. Despite this, the binary logistic regression indicates that none of the anticipated emotions predict behaviour in the presence of intention. This suggests that intention may play a mediating role in the relations between each of the anticipated emotions and TSE behaviour. To explore this possibility further, bootstrapped tests of indirect effects (PROCESS; Hayes, 2022) were performed. The results (Table 3) indicate positive indirect effects of anticipated counterfactual relief and anticipated regret on TSE behaviour through intention; *β* = .21, bootstrapped SE = .051, 95% bootstrapped CI [.13, .33]; *β* = .17, bootstrapped SE = .04, 95% bootstrapped CI [.11, .27], respectively. In addition, a negative indirect effect of anticipated temporal relief on TSE behaviour through intention was also observed; *β* = −.73, bootstrapped SE = .034, 95% bootstrapped CI [−.15, −.01]. In all tests of indirect effects, TPB variables, anticipated shame, anxiety and non‐target anticipated emotions were controlled for. The equivalent analysis without covariates is reported in Appendix S1.

**TABLE 3:** Mediation analyses testing indirect effects of anticipated counterfactual relief and anticipated regret on behaviour.

	Path coefficients				Indirect effects		
	*B* (SE)	Test statistic	LLCI	ULCI	Estimate (SE)	LLCI	ULCI
Anticipated counterfactual relief → Intention	.33 (.05)	6.22***	.23	.44			
Anticipated counterfactual relief → Behaviour	−.01 (.12)	−.10	−.24	.22			
Intention → Behaviour	.63 (.10)	6.12***	.43	.83			
Anticipated counterfactual relief → Intention → Behaviour					.21 (.05)	.13	.33
Anticipated regret → Intention	.28 (.05)	6.06***	.19	.37			
Anticipated regret → Behaviour	.075 (.10)	.78	−.11	.27			
Intention → Behaviour	.63 (.10)	6.12***	.43	.83			
Anticipated regret → Intention → Behaviour					.18 (.04)	.11	.27
Anticipated temporal relief → Intention	−.12 (.044)	−2.63**	−.20	−.029			
Anticipated temporal relief → Behaviour	−.015 (.094)	−.16	−.20	.17			
Intention → Behaviour	.63 (.10)	6.12***	.43	.83			
Anticipated temporal relief → Intention → Behaviour					−.73 (.034)	−.145	−.013

*Note*: *n* = 465. Attitudes, norms, perceived behavioural control, shame, anxiety and the non‐target anticipated emotions are included as covariates. Test statistic for path to intention is t‐statistic, test statistic for paths to behaviour are *z* statistics. Standard error and confidence intervals for indirect effects are bootstrapped. Number of bootstrapped samples = 5000.

***p < .001, **p < .01.

## DISCUSSION

By including measures of anticipated counterfactual and temporal relief as well as anticipated regret, the current study permits consideration of whether it is the negative valence of anticipated regret or its counterfactual nature which underlies its positive association with many healthful behaviours. Consistent with results reported by Lorimer et al. (2023), our results suggest that anticipated regret and anticipated counterfactual relief are positively associated with TSE, and potentially other healthful behaviours, because they are counterfactual emotions. Anticipated counterfactual relief and anticipated regret are independent, positive, predictors of intention to engage in TSE and, indirectly, TSE behaviour itself. Interestingly, anticipated temporal relief was negatively associated with intention to engage in TSE and, indirectly, TSE behaviour when other predictors were controlled for. Taken together, these results add weight to previous claims (Lorimer et al., 2023; Shepherd et al., 2017) that there is a need to consider both positive and negative emotions, especially those with counterfactual precursors, when attempting to understand the determinants of health behaviours.

The results reported herein are the first to show both counterfactual and temporal relief as significant but opposing predictors of intention to engage in a healthful behaviour and, indirectly, actual behavioural engagement. The opposing influence of anticipated temporal relief and anticipated counterfactual relief provides further evidence in support of a distinction between temporal and counterfactual relief. Previous research has shown that the two indeed come apart in terms of their precursors (e.g., Graham et al., 2023; Sweeny & Vohs, 2012), effects of their experience (Lorimer et al., 2022) and role in decision‐making (Lorimer et al., 2023). The current results add weight to the last of these findings, that anticipated temporal and counterfactual relief play different roles in decision‐making; whereas counterfactual relief was positively associated with TSE intention and, indirectly, behaviour, temporal relief was negatively associated with intention, and, indirectly, related to TSE engagement. One possibility is that anticipated temporal relief may index people's beliefs about the aversiveness of TSE. Hence, anticipated temporal relief is a negative, rather than positive, predictor of intention. To explore this possibility further, future work could assess both anticipated temporal relief and perceived aversiveness of TSE to determine whether there are changes to the observed relation between anticipated temporal relief and intention in the presence of perceived aversiveness. Ultimately, future work exploring the determinants of health behaviours should avoid confounds by distinguishing between temporal and counterfactual relief.

Interestingly, the results reported herein only partially align with those reported by Shepherd et al. (2017) who found that anticipated relief, but not anticipated regret, predicted TSE intention and past TSE behaviour. Variation in the anticipated regret measures between the two studies may account for the disparate results. Specifically, the anticipated regret measure employed within the current study prompted participants to consider the regret that they would feel if they did not perform TSE within a specific timeframe (one month), whereas Shepherd and colleagues asked participants about regret they anticipate for not ‘regularly’ performing TSE. Importantly, our results align with past research that utilized a similarly restricted timeframe (McGilligan et al., 2009). The contrast between our findings and those of Shepherd et al. suggests that anticipated regret may influence decision‐making about a particular behaviour occurring within a given timeframe but may be less influential when considering repeated or ongoing engagement in a target behaviour. Given that the anticipation of regret is contingent on simulation of the future (e.g., Sherman et al., 1995), providing more specific temporal parameters may facilitate clearer or more specific simulations that are easier to integrate into the decision‐making process. Further work is needed to explore this possibility.

The results of the current study have implications for the design of interventions to increase rates of TSE. The difference in TSE rates at Times 1 (27.9%) and 2 (53.3%) suggests that the process of assessing predictors at Time 1 may have resulted in a mere measurement effect that manifested in the Time 2 data. Measurement effects occur when behaviour is influenced by the probing of constructs believed to be precursors to the behaviour itself (see Wood et al., 2016 for review). These results suggest that relatively simple interventions, such as prompting individuals to reflect on their attitudes and intentions, or consider their future emotional states, with specific reference to regret and counterfactual relief, may have worthwhile effects on rates of TSE. Indeed, some research has attempted to utilize the assessment of anticipated regret in just this way (e.g., Godin et al., 2008, 2010; Sandberg & Conner, 2009) with varying degrees of success. Past results range from increased intention to engage in a desired behaviour (e.g., bowel screening; O'Carroll et al., 2015) to a decrease in intentions to engage in a desired behaviour (e.g., organ donation; O'Carroll et al., 2016). Psychological reactance has been offered as a possible explanation for null effects or an unintended reduction in intention when utilizing anticipated regret as a means to change behaviour. Indeed, research shows that trait psychological reactance moderated the effect of an anticipated regret intervention aimed to increase uptake of bowel screening (Hunkin et al., 2020). Theories of psychological reactance emphasize the role of negative affect (Rosenberg & Siegel, 2018) and so it is possible that the interventions that utilize the anticipation of counterfactual relief may not trigger psychological reactance in the same way as regret. Given that the study of anticipated relief is still in its infancy, further work is needed to explore its use as a means to behaviour change and any possible associations it may have with psychological reactance which may limit its usefulness.

The current study provides a replication of associations described by Lorimer et al. (2023) between anticipated regret, counterfactual relief and influenza vaccination. The diversity of the decision contexts examined across these studies suggests that anticipated regret and counterfactual relief may be of general importance in attempts to promote healthful behaviour. The observation that across two very different health‐related decisions these anticipated emotions independently predict variance in healthful behaviour suggests one reason why there have been mixed results (see Cox et al., 2014; O'Carroll et al., 2011, 2016) of attempts to use anticipated regret to increase healthful behaviour. Although anticipation of differently valenced counterfactual emotions may have additive effects within participants, equally it may be that anticipated positive counterfactual emotions may have more predictive power for some participants whereas anticipated negative counterfactual emotions have more predictive power for others. Our current study does not allow us to distinguish between these possibilities, but suggests an interesting direction for future research. For example, it could be that the predictive power of anticipated regret versus counterfactual relief differs between individuals depending on their motivation orientation, given that some people appear to be motivated by potential gains and others by potential losses (Elliot & Church, 1997; Higgins, 1997). Individual differences in approach versus avoidance orientation are known to predict differential sensitivities to emotional states (Zelenzki & Larsen, 2002). Similarly, some people may decide to be vaccinated in order to achieve the positive emotional consequences they anticipate will ensue whereas others decide to get vaccinated so as to avoid the negative emotional consequences they anticipate if they do not. Previous failures to observe associations between anticipated regret and healthful intentions or behaviour may simply reflect the proportion of participants with approach versus avoidance motivations in the sample. A related alternative possibility is that there may have been some aspect of the methods employed in these studies which primed approach versus avoidance motivations. Future work might usefully investigate these possibilities.

The results herein allow for consideration of the relation between each of the TPB constructs, and TSE intention and behaviour. Outside of the TSE context, previous meta‐analytical reviews exploring the TPB alongside anticipated regret have reported attitudes and subjective norms to be most strongly associated with behavioural intention, and intention and attitudes to be most strongly associated with actual behaviour (Sandberg & Conner, 2008). The results of the present study vary from this pattern, with attitudes having less of an influence on intention than other variables, and perceived behavioural control having greater influence. The pattern is also different for actual behaviour where perceived control is a significant, positive predictor, and attitudes are significantly, and negatively, associated with behaviour. One possibility is that the patterns observed here may be unique to the behaviour in question, TSE. Alternatively, it may be the inclusion of additional control variables such as anxiety and shame, or the inclusion of the anticipated relief variables that alters the pattern of results. Further work is needed to arbitrate between these possibilities, and to better understand any underlying relations between each of the assessed predictor variables, and TSE intention and behaviour.

There are a number of limitations that should be borne in mind when considering the results of the current study. First, the cross‐sectional nature of our analysis of intention limits our ability to make strong causal claims about the observed relations. Although our classification of variables as predictors or outcomes is theory‐based (Ajzen, 1998; Zeelenberg, 1999), and in‐line with a wealth of existing research, the concurrent assessment of predictors and outcomes will necessarily restrict confidence in any causal claims that are made. Arguably, this limitation is restricted to the Time 1 data, and a strength of the present study is that, unlike Shepherd et al. (2017), we did follow up on actual behaviour a month after the assessment of predictors rather than relying solely, as many do, on intentions assessed at Time 1. Regardless, experimental work that manipulates predictors and assesses the resultant effects on outcome variables is needed to confidently make claims about the causal nature and direction of the relations and effects observed. A second limitation associated with the design is that we only measured the anticipated emotions at one timepoint and so the stability of these variables—and their relation to intention and behaviour—over time is unclear. Given that recent work has shown that the relations between some TPB variables and intention vary with time (Hagger & Hamilton, 2024), it is possible that the relation between anticipated emotions and intention may vary in a similar fashion. To explore this further, future work could assess the anticipated emotions across multiple timepoints and over a longer period of time to establish both the consistency in the anticipation of these emotions and also their relations to pertinent outcome variables. A final limitation of the current study is that a substantial amount of variance in both TSE intention and behaviour remains unaccounted for (42% and 66%, respectively), suggesting that there are additional, unassessed factors that affect engagement in TSE. That being said, there is no behaviour for which all factors affecting intention—or behaviour itself—have been fully accounted for. Although it would be beneficial to identify as many predictors of intention and behaviour as possible, it is equally beneficial to identify predictors that account for the majority of variance in intention, as is the case in the current study, and then develop and test ways in which these predictors can be used or manipulated to increase engagement in the target behaviour.

To conclude, the results presented herein build on past research by showing that both anticipated regret and anticipated counterfactual relief are associated with TSE intention and, indirectly, behaviour. Interestingly, we provide the first evidence that temporal relief is also associated with TSE intention and, indirectly, behaviour. However, these associations function in the opposite direction, with anticipated temporal relief being a negative predictor of TSE intention and behaviour. These results indicate that both positive and negative emotions should be considered when exploring determinants of health behaviours such as TSE. Furthermore, given their positive associations, the consideration and manipulation of emotions with counterfactual precursors may be particularly important to the design of interventions that seek to increase engagement in positive health behaviours such as TSE.

## AUTHOR CONTRIBUTIONS

**Sara Lorimer:** Conceptualization; methodology; data curation; formal analysis; investigation; validation; writing – original draft; writing – review and editing. **Teresa McCormack:** Conceptualization; methodology; investigation; validation; writing – original draft; writing – review and editing; supervision; funding acquisition. **Christoph Hoerl:** Conceptualization; investigation; funding acquisition; writing – original draft; methodology; validation; writing – review and editing. **Sarah R. Beck:** Conceptualization; methodology; investigation; validation; funding acquisition; writing – original draft; writing – review and editing. **Matthew Johnston:** Conceptualization; methodology; writing – review and editing; writing – original draft; investigation; validation. **Aidan Feeney:** Conceptualization; investigation; funding acquisition; writing – original draft; methodology; validation; writing – review and editing; project administration; supervision.

## FUNDING INFORMATION

This research was funded by Grant RPG‐2018‐019 from the Leverhulme Trust.

## CONFLICT OF INTEREST STATEMENT

The authors declare no potential conflicts of interest with respect to the research, authorship and/or publication of this article.

## Supporting information


Appendix S1

## Data Availability

Research materials, data and code are available at the following link https://osf.io/yue4x/. Study and analyses were not preregistered.

## References

[bjhp12756-bib-0001] Abraham, C. , Henderson, M. , & Der, G. (2004). Cognitive impact of a research‐based school sex education programme. Psychology & Health, 19, 689–703. 10.1080/08870440410001722921

[bjhp12756-bib-0053] Abraham, C. , & Sheeran, P. (2003). Acting on intentions: The role of anticipated regret. British Journal of social psychology, 42(4), 495–511. 14715114 10.1348/014466603322595248

[bjhp12756-bib-0054] Abraham, C. , & Sheeran, P. (2004). Deciding to exercise: The role of anticipated regret. British journal of health psychology, 9(2), 269–278. 15125809 10.1348/135910704773891096

[bjhp12756-bib-0002] Ajzen, I. (1998). Models of human social behaviour and their application to health psychology. Psychology and Health, 13(4), 735–739. 10.1080/08870449808407426

[bjhp12756-bib-0003] Austin, P. C. , & Steyerberg, E. W. (2017). Events per variable (EPV) and the relative performance of different strategies for estimating the out‐of‐sample validity of logistic regression models. Statistical Methods in Medical Research, 26(2), 796–808. 10.1177/0962280214558972 25411322 PMC5394463

[bjhp12756-bib-0004] Bakker, A. B. , Buunk, B. P. , & Manstead, A. S. R. (1997). The moderating role of self‐efficacy beliefs in the relationship between anticipated feelings of regret and condom use. Journal of Applied Social Psychology, 27, 2001–2014. 10.1111/j.1559-1816.1997.tb01637.x

[bjhp12756-bib-0005] Bonner, K. E. , Vashist, K. , Abad, N. S. , Kriss, J. L. , Meng, L. , Lee, J. T. , Wilhelm, E. , Lu, P. J. , Carter, R. J. , Boone, K. , Baack, B. , Masters, N. B. , Weiss, D. , Black, C. , Huang, Q. , Vangala, S. , Albertin, C. , Szilagyi, P. G. , Brewer, N. T. , & Singleton, J. A. (2023). Behavioural and social drivers of COVID‐19 vaccination in the United States, August–November 2021. American Journal of Preventative Medicine, 64(6), 865–876. 10.1016/j.amepre.2023.01.014 PMC9874048 36775756

[bjhp12756-bib-0006] Brewer, N. T. , DeFrank, J. T. , & Gilkey, M. B. (2016). Anticipated regret and health behaviour: A meta‐analysis. Health Psychology, 35(11), 1264. 10.1037/hea0000294 27607136 PMC5408743

[bjhp12756-bib-0007] Chapman, G. B. , & Coups, E. J. (2006). Emotions and preventive health behavior: Worry, regret, and influenza vaccination. Health Psychology, 25(1), 82–90. 10.1037/0278-6133.25.1.82 16448301

[bjhp12756-bib-0008] Chong, R. I. , Leow, J. J. , Choo, Z. W. , Salada, R. , Yong, D. Z. , & Chong, Y. L. (2023). Testicular self‐examination for early detection of testicular cancer. World Journal of Urology, 41(4), 941–951. 10.1007/s00345-023-04381-4 37036497

[bjhp12756-bib-0009] Conner, M. , Sandberg, T. , McMillan, B. , & Higgins, A. (2006). Role of anticipated regret, intentions and intention stability in adolescent smoking initiation. British Journal of Health Psychology, 11(1), 85–101. 10.1348/135910705X40997 16480557

[bjhp12756-bib-0010] Cox, D. , Sturm, L. , & Cox, A. D. (2014). Effectiveness of asking anticipated regret in increasing HPV vaccination intention in mothers. Health Psychology, 33(9), 1074. 10.1037/hea0000071 24611739

[bjhp12756-bib-0011] Elliot, A. J. , & Church, M. A. (1997). A hierarchical model of approach and avoidance achievement motivation. Journal of Personality and Social Psychology, 72, 218–232. 10.1037/0022-3514.72.1.218 10234849

[bjhp12756-bib-0012] Evans, R. E. , Steptoe, A. , & Wardle, J. (2006). Testicular self‐examination: Change in rates of practice in European university students, from 13 countries, over a 10‐year period. The Journal of Men's Health & Gender, 3(4), 368–372. 10.1016/j.jmhg.2006.08.005

[bjhp12756-bib-0013] Francis, J. , Eccles, M. P. , Johnston, M. , Walker, A. E. , Grimshaw, J. M. , Foy, R. , Kaner, E. F. S. , Smith, L. , & Bonetti, D. (2004). Constructing questionnaires based on the theory of planned behaviour: A manual for health services researchers. Centre for Health Services Research, University of Newcastle upon Tyne.

[bjhp12756-bib-0014] Fung, H. , Sgaier, S. K. , & Huang, V. S. (2023). Discovery of interconnected causal drivers of COVID‐19 vaccination intentions in the US using a causal Bayesian network. Scientific Reports, 13, 6988. 10.1038/s41598-023-33745-4 37193707 PMC10188432

[bjhp12756-bib-0015] Godin, G. , Sheeran, P. , Conner, M. , Delage, G. , Germain, M. , Bélanger‐Gravel, A. , & Naccache, H. (2010). Which survey questions change behaviour? Randomized controlled trial of mere measurement interventions. Health Psychology, 29(6), 636–644. 10.1037/a0021131 20939639

[bjhp12756-bib-0016] Godin, G. , Sheeran, P. , Conner, M. , & Germain, M. (2008). Asking questions changes behaviour: Mere measurement effects in frequency of blood donation. Health Psychology, 27(2), 179–184. 10.1037/0278-6133.27.2.179 18377136

[bjhp12756-bib-0017] Graham, A. J. , McCormack, T. , Lorimer, S. , Hoerl, C. , Beck, S. R. , Johnston, M. , & Feeney, A. (2023). Relief in everyday life. Emotion, 23(7), 1844–1868. 10.1037/emo0001191 36455007

[bjhp12756-bib-0018] Hamilton, K. , & Schmidt, H. (2014). Drinking and swimming: Investigating young Australian males' intentions to engage in recreational swimming while under the influence of alcohol. Journal of Community Health, 39(1), 139–147. 10.1007/s10900-013-9751-4 23979669

[bjhp12756-bib-0056] Hagger, M. S. , & Hamilton, K. (2024). Longitudinal tests of the theory of planned behaviour: A meta‐analysis. European Review of Social Psychology, 35(1), 198–254.

[bjhp12756-bib-0057] Hayes, A. F. , (2022). Introduction to mediation, moderation, and conditional process analysis: A regression‐based approach (vol. 3). The Guilford Press.

[bjhp12756-bib-0019] Higgins, E. T. (1997). Beyond pleasure and pain. American Psychologist, 52, 1280–1300. 10.1037/0003-066X.52.12.1280 9414606

[bjhp12756-bib-0020] Hoerl, C. (2015). Tense and the psychology of relief. Topoi, 34(1), 217–231. 10.1007/s11245-013-9226-3

[bjhp12756-bib-0021] Hunkin, H. , Turnbull, D. , & Zajac, I. T. (2020). Considering anticipated regret may reduce colorectal cancer screening intentions: A randomised controlled trial. Psychology & Health, 35(5), 555–572. 10.1080/08870446.2019.1649407 31403327

[bjhp12756-bib-0022] Huyghe, E. , Plante, P. , & Thonneau, P. F. (2007). Testicular cancer variations in time and space in Europe. European Urology, 51(3), 621–628. 10.1016/j.eururo.2006.08.024 16963178

[bjhp12756-bib-0058] Iyigun, E. , Tastan, S. , Ayhan, H. , Kose, G. , & Acikel, C. (2016). Validity and reliability analysis of the planned behavior theory scale related to the testicular self‐examination in a Turkish context. Postgraduate Medicine, 128(5), 496–501. 27130481 10.1080/00325481.2016.1182872

[bjhp12756-bib-0024] Landman, J. (1993). Regret: The persistence of the possible. Oxford University Press.

[bjhp12756-bib-0025] Lazuras, L. , Chatzipolychroni, E. , Rodafinos, A. , & Eiser, J. R. (2012). Social cognitive predictors of smoking cessation intentions among smoker employees: The roles of anticipated regret and social norms. Addictive Behaviors, 37(3), 339–341. 10.1016/j.addbeh.2011.11.008 22154507

[bjhp12756-bib-0026] Loomes, G. , & Sugden, R. (1982). Regret theory: An alternative theory of rational choice under uncertainty. The Economic Journal, 92(368), 805–824. 10.2307/2232669

[bjhp12756-bib-0027] Lorimer, S. , McCormack, T. , Hoerl, C. , Johnston, M. , Beck, S. R. , & Feeney, A. (2023). Do both anticipated relief and anticipated regret predict decisions about influenza vaccination? British Journal of Health Psychology, 29, 134–148. 10.1111/bjhp.12691 37722923

[bjhp12756-bib-0028] Lorimer, S. , McCormack, T. , Jaroslawska, A. J. , Hoerl, C. , Beck, S. R. , Johnston, M. , & Feeney, A. (2022). From Brexit to Biden: What responses to national outcomes tell us about the nature of relief. Social Psychological and Personality Science, 13(7), 1095–1104. 10.1177/19485506211066712

[bjhp12756-bib-0029] McClenahan, C. , Shevlin, M. , Adamson, G. , Bennett, C. , & O'Neill, B. (2007). Testicular self‐examination: A test of the health belief model and the theory of planned behaviour. Health Education Research, 22(2), 272–284. 10.1093/her/cyl076 16885203

[bjhp12756-bib-0030] McGilligan, C. , McClenahan, C. , & Adamson, G. (2009). Attitudes and intentions to performing testicular self‐examination: Utilizing an extended theory of planned behaviour. Journal of Adolescent Health, 44(4), 404–406. 10.1016/j.jadohealth.2008.08.018 19306801

[bjhp12756-bib-0031] Miller, A. S. , Aisenbrey, S. , & Kimmel, D. M. (2022). Awareness and performance of testicular self‐examinations: An analysis of social and cultural barriers to cancer screenings in a US orthodox Jewish community. Journal of Religion and Health, 61(6), 4398–4419. 10.1007/s10943-022-01623-4 35913643

[bjhp12756-bib-0032] Nichol, K. L. , Margolis, K. L. , Lind, A. , Murdoch, M. , McFadden, R. , Hauge, M. , Magnan, S. , & Drake, M. (1996). Side effects associated with influenza vaccination in healthy working adults: A randomized, placebo‐controlled trial. Archives of Internal Medicine, 156, 1546–1550. 10.1001/archinte.1996.00440130090009 8687262

[bjhp12756-bib-0033] O'Carroll, R. E. , Chambers, J. A. , Brownlee, L. , Libby, G. , & Steele, R. J. C. (2015). Anticipated regret to increase uptake of colorectal cancer screening (ARTICS): A randomized controlled trial. Social Science and Medicine, 142, 118–127. 10.1016/j.socscimed.2015.07.026 26301484 PMC4576211

[bjhp12756-bib-0034] O'Carroll, R. E. , Dryden, J. , Hamilton‐Barclay, T. , & Ferguson, E. (2011). Anticipated regret and organ donor registration—A pilot study. Health Psychology, 30(5), 661–664. 10.1037/a0024182 21644807

[bjhp12756-bib-0035] O'Carroll, R. E. , Shepherd, L. , Hayes, P. C. , & Ferguson, E. (2016). Anticipated regret and organ donor registration: A randomized controlled trial. Health Psychology, 35(11), 1169–1177. 10.1037/hea0000363 27280372

[bjhp12756-bib-0036] Park, J. S. , Kim, J. K. , Elghiaty, A. , & Ham, W. S. (2018). Recent global trends in testicular cancer incidence and mortality. Medicine, 97(37), e12390. 10.1097/MD.0000000000012390 30213007 PMC6155960

[bjhp12756-bib-0037] Peltzer, K. , & Pengpid, S. (2015). Knowledge, attitudes and practice of testicular self‐examination among male university students from Bangladesh, Madagascar, Singapore, South Africa and Turkey. Asian Pacific Journal of Cancer Prevention, 16(11), 4741–4743. 10.7314/APJCP.2014.16.11.4741 26107234

[bjhp12756-bib-0038] Rosenberg, B. D. , & Siegel, J. T. (2018). A 50‐year review of psychological reactance theory: Do not read this article. Motivation Science, 4(4), 281. 10.1037/mot0000091

[bjhp12756-bib-0039] Rothman, A. J. , & Sheeran, P. (2021). The operating conditions framework: Integrating mechanisms and moderators in health behaviour interventions. Health Psychology, 40(12), 845. 10.1037/hea0001026 32914997

[bjhp12756-bib-0059] Sandberg, T. , & Conner, M. (2008). Anticipated regret as an additional predictor in the theory of planned behaviour: A meta‐analysis. British Journal of Social Psychology, 47(4), 589–606. 18039428 10.1348/014466607X258704

[bjhp12756-bib-0040] Sandberg, T. , & Conner, M. (2009). A mere measurement effect for anticipated regret: Impacts on cervical screening attendance. British Journal of Social Psychology, 48, 221–236. 10.1348/014466608X347001 18793492

[bjhp12756-bib-0041] Shanmugalingam, T. , Soultati, A. , Chowdhury, S. , Rudman, S. , & Van Hemelrijck, M. (2013). Global incidence and outcome of testicular cancer. Clinical Epidemiology, 5, 417. 10.2147/CLEP.S34430 24204171 PMC3804606

[bjhp12756-bib-0042] Shepherd, L. , Watt, C. , & Lovell, B. (2017). The role of social–cognitive and emotional factors on testicular self‐examination. Psycho‐Oncology, 26(1), 53–59. 10.1002/pon.4097 26890315

[bjhp12756-bib-0043] Sherman, S. J. , McConnell, A. R. , Roese, N. J. , & Olson, J. M. (1995). Dysfunctional implications of counterfactual thinking: When alternatives to reality fail us. In N. J. Roese & J. M. Olson (Eds.), What might have been: The social psychology of counterfactual thinking (pp. 199–231). Psychology Press.

[bjhp12756-bib-0055] Steinmetz, H. , Knappstein, M. , Ajzen, I. , Schmidt, P. , & Kabst, R. (2016). How effective are behavior change interventions based on the theory of planned behavior?. Zeitschrift für Psychologie.

[bjhp12756-bib-0044] Sweeny, K. , & Vohs, K. D. (2012). On near misses and completed tasks: The nature of relief. Psychological Science, 23(5), 464–468. 10.1177/0956797611434590 22477104

[bjhp12756-bib-0045] Ugurlu, Z. , Akkuzu, G. , Karahan, A. , Beder, A. , Dogan, N. , Okdem, S. , & Kav, S. (2011). Testicular cancer awareness and testicular self‐examination among university students. Asian Pacific Journal of Cancer Prevention, 12(3), 695–698. 21627366

[bjhp12756-bib-0046] United States Preventative Services Task Force . (2011). Screening for testicular cancer: U.S. Preventative Services Task Force reaffirmation recommendation statement. Annals of Internal Medicine, 154(7), 483–486. 10.7326/0003-4819-154-7-201104050-00006 21464350

[bjhp12756-bib-0047] Wood, C. , Conner, M. , Miles, E. , Sandberg, T. , Taylor, N. , Godin, G. , & Sheeran, P. (2016). The impact of asking intention or self‐prediction questions on subsequent behaviour: A meta‐analysis. Personality and Social Psychology Review, 20(3), 245–268. 10.1177/1088868315592334 26162771 PMC4931712

[bjhp12756-bib-0048] Zeelenberg, M. (1999). Anticipated regret expected feedback and behavioural decision making. Journal of Behavioural Decision Making, 12(2), 93–106. 10.1002/(SICI)1099-0771(199906)12:2<93::AID-BDM311>3.0.CO;2-S

[bjhp12756-bib-0049] Zeelenberg, M. , & Pieters, R. (2007). A theory of regret regulation 1.0. Journal of Consumer Psychology, 17(1), 3–18. 10.1207/s15327663jcp1701_3 PMC2435165 18568095

[bjhp12756-bib-0050] Zelenzki, J. M. , & Larsen, R. J. (2002). Susceptibility to affect: A comparison of three personality taxonomies. Journal of Personality, 67(5), 761–791. 10.1111/1467-6494.00072 10540757

[bjhp12756-bib-0051] Ziarnowski, K. L. , Brewer, N. T. , & Weber, B. (2009). Present choices, future outcomes: Anticipated regret and HPV vaccination. Preventive Medicine, 48, 411–414. 10.1016/j.ypmed.2008.10.006 18996144

[bjhp12756-bib-0052] Znaor, A. , Lortet‐Tieulent, J. , Jemal, A. , & Bray, F. (2014). International variations and trends in testicular cancer incidence and mortality. European Urology, 65(6), 1095–1106. 10.1016/j.eururo.2013.11.004 24268506

