# Supplementary material for: Testicular self‐examination: The role of anticipated relief and anticipated regret
Source: Br J Health Psychol. 2024 Sep 29;30(1):e12756. doi: 10.1111/bjhp.12756 (PMC11586803; doi:10.1111/bjhp.12756)
Supplement: Supplementary file 1 — Appendix S1 [file BJHP-30-0-s001.docx]

**Comparison of questions to Shepherd et al., (2017)**

Table 1. Comparison of questions used by Shepherd et al., (2017) and those used in the current study.

|  | Shepherd et al., (2017) | Current study | Justification of change |
| --- | --- | --- | --- |
| Anticipated regret | 1. ‘To what extent are you likely to feel regret if you did not check your testicles for lumps regularly?’ 2. ‘To what extent are you likely to feel regret for not regularly checking your testicles?’ 3. ‘To what extent are you likely to feel regret if you did not regularly perform testicular self-examination?’ | 1. ‘If I did not perform a testicular self-examination within the next month, rather than deciding to do it, I would feel regret.’ 2. ‘If I did not perform a testicular self-examination within the next month, rather than deciding to do it, I would feel upset.’ | - Timeframe is constrained to duration of follow-up period. - Counterfactual is provided [prefactual: if did not perform, counterfactual: did perform] |
| Anticipated relief | 1. ‘To what extent would performing testicular self-examination make you feel relieved?’ 2. ‘To what extent would performing testicular self-examination make you feel reassured?’ 3. ‘To what extent would performing testicular self-examination make you feel at ease?’ | Counterfactual relief   1. ‘If I performed a testicular self-examination within the next month, rather than deciding not to, I would feel relieved.’ 2. ‘If I performed a testicular self-examination within the next month, rather than deciding not to, I would feel glad.’   Temporal relief   1. ‘If I performed a testicular self-examination within the next month, I would feel relieved once it was over.’ 2. ‘If I performed a testicular self-examination within the next month, I would feel glad once it was over.’ | - Distinguish between counterfactual and temporal relief. - Timeframe is constrained to duration of follow-up period. - Given the related precursors, regret and counterfactual relief are strongly aligned in terms of phrasing. - Counterfactual is provided for counterfactual relief [prefactual: if did perform, counterfactual: did not perform] |

**Time 1 Questionnaire**

Q1 Please select your **age** from the dropdown list

- Prefer not to say (1)

Q2 What is the highest degree or level of school you have completed? If you are currently enrolled in education, please indicate the highest degree/level of education you have obtained.

- Professional or doctorate degree (e.g., MD, PhD) (1)
- Masters degree (e.g., MDc, MA, MBA) or postgraduate diploma (e.g., PGCE) or equivalent (e.g., Level 7 NVQ) (2)
- Bachelor's degree (e.g., BA, BSc) or equivalent (e.g., Level 6 NVQ) (3)
- Higher national certificate (HNC) or diploma (HND) or equivalent (e.g., Level 4 or 5 NVQ) (4)
- A and AS Level or equivalent (e.g., Level 3 NVQ) (5)
- GCSE grades A*-C or equivalent (e.g., Level 2 NVQ) (6)
- GCSE grades D-G or equivalent (e.g., Level 1 NVQ) (7)
- No qualifications (8)
- I prefer not to say (9)

Q3 Are you currently in a relationship?

- Yes (1)
- No (2)

Using the scales provided, please indicate the extent to which you agree with the following statements:

Q4 *Performing a testicular self-examination within the next month would be…*

|  | harmful | beneficial |
| --- | --- | --- |

|  | 1 | 2 | 3 | 4 | 5 | 6 | 7 |
| --- | --- | --- | --- | --- | --- | --- | --- |

| () | 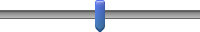 |
| --- | --- |

Q5

|  | good | bad |
| --- | --- | --- |

|  | 1 | 2 | 3 | 4 | 5 | 6 | 7 |
| --- | --- | --- | --- | --- | --- | --- | --- |

| () | 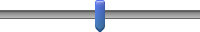 |
| --- | --- |

Q6

|  | pleasant | unpleasant |
| --- | --- | --- |

|  | 1 | 2 | 3 | 4 | 5 | 6 | 7 |
| --- | --- | --- | --- | --- | --- | --- | --- |

| () | 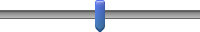 |
| --- | --- |

Q7

|  | useless | useful |
| --- | --- | --- |

|  | 1 | 2 | 3 | 4 | 5 | 6 | 7 |
| --- | --- | --- | --- | --- | --- | --- | --- |

| () | 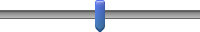 |
| --- | --- |

Using the scales provided, please indicate the extent to which you agree with the following:

Q8 *Most people who are important to me think I should perform a testicular self-examination within the next month*

|  | Strongly disagree | Strongly agree |
| --- | --- | --- |

|  | 1 | 2 | 3 | 4 | 5 | 6 | 7 |
| --- | --- | --- | --- | --- | --- | --- | --- |

| () | 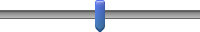 |
| --- | --- |

Q9 *People who are important to me want me to perform a testicular self-examination within the next month*

|  | Strongly disagree | Strongly agree |
| --- | --- | --- |

|  | 1 | 2 | 3 | 4 | 5 | 6 | 7 |
| --- | --- | --- | --- | --- | --- | --- | --- |

| () | 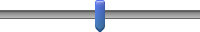 |
| --- | --- |

Q10 *It is expected of me that I will perform a testicular self-examination within the next month*

|  | Strongly disagree | Strongly agree |
| --- | --- | --- |

|  | 1 | 2 | 3 | 4 | 5 | 6 | 7 |
| --- | --- | --- | --- | --- | --- | --- | --- |

| () | 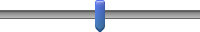 |
| --- | --- |

Q11 *I feel under social pressure to perform a testicular self-examination within the next month*

|  | Strongly disagree | Strongly agree |
| --- | --- | --- |

|  | 1 | 2 | 3 | 4 | 5 | 6 | 7 |
| --- | --- | --- | --- | --- | --- | --- | --- |

| () | 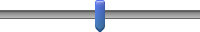 |
| --- | --- |

Using the scales provided, please indicate the extent to which you agree with the following statements:

Q12 *I am confident that I could perform a testicular self-examination within the next month*

|  | Strongly disagree | Strongly agree |
| --- | --- | --- |

|  | 1 | 2 | 3 | 4 | 5 | 6 | 7 |
| --- | --- | --- | --- | --- | --- | --- | --- |

| () | 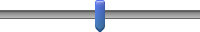 |
| --- | --- |

Q13 *It would be easy for me to perform a testicular self-examination within the next month*

|  | Strongly disagree | Strongly agree |
| --- | --- | --- |

|  | 1 | 2 | 3 | 4 | 5 | 6 | 7 |
| --- | --- | --- | --- | --- | --- | --- | --- |

| () | 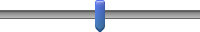 |
| --- | --- |

Q14 *The decision to perform a testicular self-examination within the next month is beyond my control*

|  | Strongly disagree | Strongly agree |
| --- | --- | --- |

|  | 1 | 2 | 3 | 4 | 5 | 6 | 7 |
| --- | --- | --- | --- | --- | --- | --- | --- |

| () | 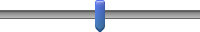 |
| --- | --- |

Q15 *Whether I perform a testicular self-examination within the next month is entirely up to me*

|  | Strongly disagree | Strongly agree |
| --- | --- | --- |

|  | 1 | 2 | 3 | 4 | 5 | 6 | 7 |
| --- | --- | --- | --- | --- | --- | --- | --- |

| () | 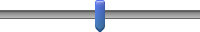 |
| --- | --- |

Using the scales provided, please indicate your response to the following questions:

Q16 *If I performed a testicular self-examination within the next month, rather than deciding not to, I would feel relieved.*

|  | Strongly disagree | Strongly agree |
| --- | --- | --- |

|  | 1 | 2 | 3 | 4 | 5 | 6 | 7 |
| --- | --- | --- | --- | --- | --- | --- | --- |

| () | 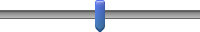 |
| --- | --- |

Q17 *If I performed a testicular self-examination within the next month, rather than deciding not to, I would feel glad.*

|  | Strongly disagree | Strongly agree |
| --- | --- | --- |

|  | 1 | 2 | 3 | 4 | 5 | 6 | 7 |
| --- | --- | --- | --- | --- | --- | --- | --- |

| () | 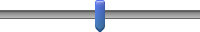 |
| --- | --- |

Using the scales provided, please indicate your response to the following questions:

Q18 *If I performed a testicular self-examination within the next month, I would feel relieved once it was over.*

|  | Strongly disagree | Strongly agree |
| --- | --- | --- |

|  | 1 | 2 | 3 | 4 | 5 | 6 | 7 |
| --- | --- | --- | --- | --- | --- | --- | --- |

| () | 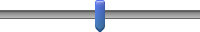 |
| --- | --- |

Q19 *If I performed a testicular self-examination within the next month, I would feel glad once it was over.*

|  | Strongly disagree | Strongly agree |
| --- | --- | --- |

|  | 1 | 2 | 3 | 4 | 5 | 6 | 7 |
| --- | --- | --- | --- | --- | --- | --- | --- |

| () | 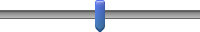 |
| --- | --- |

Using the scales provided, please indicate your response to the following questions:

Q20 *If I did not perform a testicular self-examination within the next month, rather than deciding to do it, I would feel regret.*

|  | Strongly disagree | Strongly agree |
| --- | --- | --- |

|  | 1 | 2 | 3 | 4 | 5 | 6 | 7 |
| --- | --- | --- | --- | --- | --- | --- | --- |

| () | 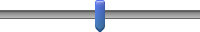 |
| --- | --- |

Q21 *If I did not perform a testicular self-examination within the next month, rather than deciding to do it, I would feel upset.*

|  | Strongly disagree | Strongly agree |
| --- | --- | --- |

|  | 1 | 2 | 3 | 4 | 5 | 6 | 7 |
| --- | --- | --- | --- | --- | --- | --- | --- |

| () | 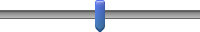 |
| --- | --- |

Using the scales provided, please indicate your response to the following questions:

Q22 *To what extent would you feel shame if you were diagnosed with testicular cancer?*

|  | not at all | extremely |
| --- | --- | --- |

|  | 1 | 2 | 3 | 4 | 5 | 6 | 7 |
| --- | --- | --- | --- | --- | --- | --- | --- |

| () | 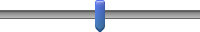 |
| --- | --- |

Q23 *To what extent would you feel embarrassed if you were diagnosed with testicular cancer?*

|  | not at all | extremely |
| --- | --- | --- |

|  | 1 | 2 | 3 | 4 | 5 | 6 | 7 |
| --- | --- | --- | --- | --- | --- | --- | --- |

| () | 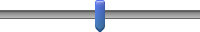 |
| --- | --- |

Q24 *To what extent would you feel humiliated if you were diagnosed with testicular cancer?*

|  | not at all | extremely |
| --- | --- | --- |

|  | 1 | 2 | 3 | 4 | 5 | 6 | 7 |
| --- | --- | --- | --- | --- | --- | --- | --- |

| () | 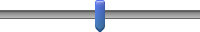 |
| --- | --- |

Q25 *To what extent would you feel ashamed if you had to meet with a medical professional to discuss testicular cancer?*

|  | not at all | extremely |
| --- | --- | --- |

|  | 1 | 2 | 3 | 4 | 5 | 6 | 7 |
| --- | --- | --- | --- | --- | --- | --- | --- |

| () | 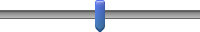 |
| --- | --- |

Q26 *To what extent would you feel embarrassed if you had to meet with a medical professional to discuss testicular cancer?*

|  | not at all | extremely |
| --- | --- | --- |

|  | 1 | 2 | 3 | 4 | 5 | 6 | 7 |
| --- | --- | --- | --- | --- | --- | --- | --- |

| () | 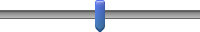 |
| --- | --- |

Q27 *To what extent would you feel humiliated if you had to meet with a medical professional to discuss testicular cancer?*

|  | not at all | extremely |
| --- | --- | --- |

|  | 1 | 2 | 3 | 4 | 5 | 6 | 7 |
| --- | --- | --- | --- | --- | --- | --- | --- |

| () | 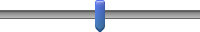 |
| --- | --- |

Q28 *To what extent would you feel ashamed if you had a testicle removed?*

|  | not at all | extremely |
| --- | --- | --- |

|  | 1 | 2 | 3 | 4 | 5 | 6 | 7 |
| --- | --- | --- | --- | --- | --- | --- | --- |

| () | 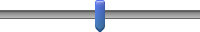 |
| --- | --- |

Q29 *To what extent would you feel embarrassed if you had a testicle removed?*

|  | not at all | extremely |
| --- | --- | --- |

|  | 1 | 2 | 3 | 4 | 5 | 6 | 7 |
| --- | --- | --- | --- | --- | --- | --- | --- |

| () | 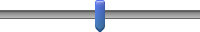 |
| --- | --- |

Q30 *To what extent would you feel humiliated if you had a testicle removed?*

|  | not at all | extremely |
| --- | --- | --- |

|  | 1 | 2 | 3 | 4 | 5 | 6 | 7 |
| --- | --- | --- | --- | --- | --- | --- | --- |

| () | 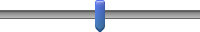 |
| --- | --- |

Using the scales provided, please indicate your response to the following questions:

Q31 *To what extent are you afraid that you may have testicular cancer?*

|  | not at all | extremely |
| --- | --- | --- |

|  | 1 | 2 | 3 | 4 | 5 | 6 | 7 |
| --- | --- | --- | --- | --- | --- | --- | --- |

| () | 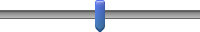 |
| --- | --- |

Q32 *To what extent are you worried that you may have testicular cancer?*

|  | not at all | extremely |
| --- | --- | --- |

|  | 1 | 2 | 3 | 4 | 5 | 6 | 7 |
| --- | --- | --- | --- | --- | --- | --- | --- |

| () | 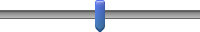 |
| --- | --- |

Q33 *To what extent are you anxious that you may have testicular cancer?*

|  | not at all | extremely |
| --- | --- | --- |

|  | 1 | 2 | 3 | 4 | 5 | 6 | 7 |
| --- | --- | --- | --- | --- | --- | --- | --- |

| () | 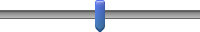 |
| --- | --- |

Q34 *To what extent are you afraid that sometimes, medical procedures are performed on people without their consent?*

|  | not at all | extremely |
| --- | --- | --- |

|  | 1 | 2 | 3 | 4 | 5 | 6 | 7 |
| --- | --- | --- | --- | --- | --- | --- | --- |

| () | 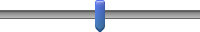 |
| --- | --- |

Q35 *To what extent are you worried that sometimes, medical procedures are performed on people without their consent?*

|  | not at all | extremely |
| --- | --- | --- |

|  | 1 | 2 | 3 | 4 | 5 | 6 | 7 |
| --- | --- | --- | --- | --- | --- | --- | --- |

| () | 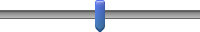 |
| --- | --- |

Q36 *To what extent are you anxious that sometimes, medical procedures are performed on people without their consent?*

|  | not at all | extremely |
| --- | --- | --- |

|  | 1 | 2 | 3 | 4 | 5 | 6 | 7 |
| --- | --- | --- | --- | --- | --- | --- | --- |

| () | 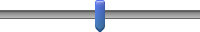 |
| --- | --- |

Q37 *To what extent are you afraid that you may have a testicle removed if you found a lump?*

|  | not at all | extremely |
| --- | --- | --- |

|  | 1 | 2 | 3 | 4 | 5 | 6 | 7 |
| --- | --- | --- | --- | --- | --- | --- | --- |

| () | 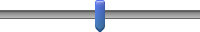 |
| --- | --- |

Q38 *To what extent are you worried that you may have a testicle removed if you found a lump?*

|  | not at all | extremely |
| --- | --- | --- |

|  | 1 | 2 | 3 | 4 | 5 | 6 | 7 |
| --- | --- | --- | --- | --- | --- | --- | --- |

| () | 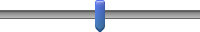 |
| --- | --- |

Q39 *To what extent are you anxious that you may have a testicle removed if you found a lump?*

|  | not at all | extremely |
| --- | --- | --- |

|  | 1 | 2 | 3 | 4 | 5 | 6 | 7 |
| --- | --- | --- | --- | --- | --- | --- | --- |

| () | 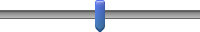 |
| --- | --- |

Using the scales provided, please indicate your response to the following questions:

Q40 *I intend to perform a testicular self-examination in the next month*

|  | strongly disagree | strongly agree |
| --- | --- | --- |

|  | 1 | 2 | 3 | 4 | 5 | 6 | 7 |
| --- | --- | --- | --- | --- | --- | --- | --- |

| () | 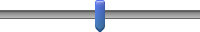 |
| --- | --- |

Q41 *I expect to perform a testicular self-examination in the next month*

|  | strongly disagree | strongly agree |
| --- | --- | --- |

|  | 1 | 2 | 3 | 4 | 5 | 6 | 7 |
| --- | --- | --- | --- | --- | --- | --- | --- |

| () | 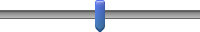 |
| --- | --- |

Q42 *I want to perform a testicular self-examination in the next month*

|  | strongly disagree | strongly agree |
| --- | --- | --- |

|  | 1 | 2 | 3 | 4 | 5 | 6 | 7 |
| --- | --- | --- | --- | --- | --- | --- | --- |

| () | 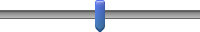 |
| --- | --- |

Q43 To your knowledge, how often should testicular self-examination be performed?

- Every day (1)
- Every week (2)
- Every month (3)
- Every three months (4)
- Every six months (5)
- Never (6)

Q44 Have you performed a testicular self-examination in the last month?

- Yes (1)
- No (2)

**Time 2 Questionnaire**

Q1 Have you performed a testicular self-examination in the last month?

- Yes
- No

|  | **M (SD)** | **1** | **2** | **3** | **4** | **5** | **6** | **7** | **8** | **9** |
| --- | --- | --- | --- | --- | --- | --- | --- | --- | --- | --- |
| 1. ATR | 4.97 (1.39) | -- |  |  |  |  |  |  |  |  |
| 1. ACF | 4.87 (1.40) | .42*** | -- |  |  |  |  |  |  |  |
| 1. AReg | 3.42 (1.56) | .31*** | .52*** | -- |  |  |  |  |  |  |
| 1. Attitudes | 6.0 (1.07) | .095* | .38*** | .16*** | -- |  |  |  |  |  |
| 1. Subjective norms | 3.44 (1.38) | .20*** | .45*** | .51*** | .29*** | -- |  |  |  |  |
| 1. Perceived control | 6.08 (.95) | -.002 | .32*** | -.002 | .47*** | .21*** | -- |  |  |  |
| 1. Anxiety | 3.10 (1.13) | .17*** | .10* | .28*** | -.090* | .14*** | -.22*** | -- |  |  |
| 1. Shame | 3.03 (1.57) | .066 | -.10* | .032 | -.22*** | -.084* | -.29*** | .47*** | -- |  |
| 1. Intention | 4.89 (1.75) | .13** | .59*** | .48*** | .43*** | .55*** | .48*** | .11** | -.17*** | -- |
| 1. Examination | NA | .046 | .27*** | .28*** | .12* | .30*** | .27*** | .020 | -.088 | .49*** |

*Table 2. Means, standard deviations, and intercorrelations of key variables.*

*Note: All variables were measured on a scale from 1-7, except examination which is binary coded with non-examination as reference. N = 567 for all variables except associations with Examination where N = 465. *** p < .001, ** p < .01, * p < .05.*

Table 3. Mediation analyses testing indirect effects of anticipated counterfactual relief and anticipated regret on behaviour.

|  | Path coefficients | | | | Indirect effects | | |
| --- | --- | --- | --- | --- | --- | --- | --- |
|  | B (SE) | Test statistic | LLCI | ULCI | Estimate (SE) | LLCI | ULCI |
| Anticipated counterfactual relief 🡪 Intention | .74 (.05) | 15.44*** | .65 | .84 |  |  |  |
| Anticipated counterfactual relief 🡪 Behaviour | -.02 (.10) | -.23 | -.21 | .17 |  |  |  |
| Intention 🡪 Behaviour | .70 (.09) | 8.17*** | .53 | .87 |  |  |  |
| Anticipated counterfactual relief 🡪 Intention 🡪 Behaviour |  |  |  |  | .52 (.07) | .39 | .68 |
| Anticipated regret 🡪 Intention | .57 (.05) | 12.72*** | .49 | .66 |  |  |  |
| Anticipated regret 🡪 Behaviour | .06 (.08) | .81 | -.09 | .22 |  |  |  |
| Intention 🡪 Behaviour | .66 (.08) | 8.19*** | .50 | .82 |  |  |  |
| Anticipated regret 🡪 Intention 🡪 Behaviour |  |  |  |  | .38 (.05) | .29 | .50 |
| Anticipated temporal relief 🡪 Intention | -.16 (.06) | 2.67** | .04 | .27 |  |  |  |
| Anticipated temporal relief 🡪 Behaviour | -.03 (.08) | -.36 | -.19 | .13 |  |  |  |
| Intention 🡪 Behaviour | .69 (.07) | 9.37*** | .55 | .84 |  |  |  |
| Anticipated temporal relief 🡪 Intention 🡪 Behaviour |  |  |  |  | .11 (.05) | .02 | .21 |

*Note: n = 465. No covariates are included in the above analysis. Test statistic for path to intention is t-statistic, test statistic for paths to behaviour are z statistics. Standard error and confidence intervals for indirect effects are bootstrapped. Number of bootstrapped samples = 5000. *** p < .001, ** p < .01.*
